# Design of Knitted Fabrics with Biomimetic Bird Feather Hierarchical Structures for Thermal and Moisture Adaptation in Outdoor Environments for the Elderly

**DOI:** 10.3390/biomimetics11060364

**Published:** 2026-05-22

**Authors:** Yuan Shu, Panpan Li, Yihan Wang, Yangyang Wei

**Affiliations:** 1 Architecture and Design College, Nanchang University, Nanchang 330031, China; 2 School of Creative Deign, Wuhan Business University, Wuhan 430056, China

**Keywords:** biomimetics, feather structure, knitted structure, gray near-optimal model, outdoor clothing for the elderly

## Abstract

Bird feathers possess functions such as water resistance, thermal insulation, and air permeability, providing inspiration for the design of functional fabrics. Based on the functional differentiation of different feather regions and the structural constraints associated with these functions, this study selected down feathers, feather vanes, hooklets, and fluffy feather filament node structures as biomimetic prototypes. Four biomimetic knitted structures were designed for outdoor environments with significant temperature fluctuations and for the thermo-moisture comfort needs of older adults. Through macro- and micro-structural feature extraction, three-dimensional modeling, and experimental testing, a multi-parameter evaluation system covering water resistance, thermal resistance, thermal insulation rate, air permeability, moisture vapor transmission, and moisture management was established to systematically evaluate the thermo-moisture regulation performance of the fabrics. The results showed that each structure exhibited distinct performance advantages: Structure 1 demonstrated the best thermal insulation performance; Structure 2 showed relatively superior water resistance and outstanding air permeability; Structure 4 exhibited relatively superior moisture vapor transmission and moisture management performance; and Structure 3 achieved the highest gray relational optimality value, indicating a relatively balanced thermo-moisture regulation capability. Among all performance indicators, air permeability showed the highest correlation with the knitted structures. Based on these results, and considering regional differences in heat generation and sweating across different body parts of older adults, this study further explored zonal application strategies for elderly outdoor clothing to improve wearing comfort and functionality under environments with fluctuating thermal conditions.

## 1. Introduction

With the acceleration of global population aging, the demand among elderly individuals for daily leisure, walking, and light outdoor activities continues to increase. Compared with younger populations, the elderly exhibit significant differences in thermoregulation, regional sweat distribution, and environmental adaptability. Previous studies have shown that aging is accompanied by a series of physiological changes related to thermal regulation, including a decline in basal metabolic rate, reduced skin blood flow regulation, and diminished sweating response, all of which lead to a decreased thermoregulatory capacity in elderly individuals [1,2,3,4]. In the evaluation of textile thermal–moisture comfort, parameters such as air permeability, moisture vapor transmission, thermal resistance, and moisture transport properties are commonly used to characterize the regulation of heat and moisture transfer between the human body and the environment [5,6,7]. Among these, higher thermal resistance helps reduce heat loss in cold conditions, while good air permeability and moisture permeability contribute to maintaining a stable microclimate at the skin surface and prevent heat and moisture accumulation. Therefore, in the design of functional clothing for the elderly, it is essential to balance thermal insulation and heat dissipation to meet their specific thermal–moisture comfort requirements. As a result, higher demands are placed on outdoor clothing in terms of thermal insulation, water resistance, air permeability, moisture permeability, and hygroscopic performance. How to improve the overall thermal–moisture comfort of elderly outdoor clothing through fabric structural design has thus become an important issue in the field of functional apparel design.

Knitted fabrics, as an important component of modern textiles, have long been a key focus in textile science, particularly in terms of structural design and performance optimization [8]. Biomimetics, as an interdisciplinary field bridging nature and technological innovation, has demonstrated significant application value across various domains [9,10,11]. In functional materials and textile design, many researchers have attempted to translate biological structural features into fabric design strategies to enhance material performance [12,13]. Previous studies have shown that biomimetic design can improve textile performance by drawing inspiration from efficient natural structures that enable functions such as water resistance, thermal insulation, and breathability. For example, Shateri-Khalilabad et al. [14], inspired by the superhydrophobic properties of lotus leaves, developed a novel coating method to improve the hydrophobicity of cotton textiles. Their results showed that the coating endowed cotton fabrics with a superhydrophobic surface as well as excellent ultraviolet-blocking performance. Wu Mingrui et al. [15], based on the core–shell structure of polar bear hair, developed a new type of fiber for sweater production, achieving thermal insulation performance superior to that of down jackets. Jayamaha et al. [16], inspired by the hierarchical structure of bird-of-paradise feathers, produced an ultra-black fabric from merino wool with an average reflectance of 0.13%, demonstrating potential applications in camouflage and optics. Other researchers have also approached the development of smart textiles from a biomimetic perspective, imitating natural phenomena and proposing corresponding design strategies, thereby promoting the advancement of the textile industry [17].

Due to their unique hierarchical structures and multifunctional properties, bird feathers have become important models for biomimetic research in various fields. In aerospace, Hoffmann et al. [18] systematically explained the design principles of feather hierarchical structures and proposed translating them into intelligent composite materials with embedded sensing functions to improve the environmental adaptability of aircraft. In terms of aerodynamic optimization, Murayama et al. [19], inspired by bird contour feathers, found that flexible flaps can effectively enhance the aerodynamic robustness of fixed-wing unmanned aerial vehicles in disturbed airflow by suppressing large-scale vortex shedding. Tu et al. [20] fabricated biomimetic feathers with hierarchical vane structures using multi-scale 3D printing and embedded piezoresistive and piezoelectric sensors, enabling real-time sensing of feather vibration and aerodynamic forces. In the field of textile materials, Wang Jianping et al. [21] developed eight knitted fabric structures inspired by bird feathers, which was applied to ski thermal pants to improve thermo-moisture comfort, providing new ideas for functional clothing design. From the perspective of structural mechanisms, the excellent functional properties of feathers are derived not only from their macroscopic morphology but are also closely related to their micro-structural features. Studies have shown that, during vibration, the vane structure dissipates energy through friction generated by the interlocking and relative sliding of barbules, thereby endowing feathers with effective damping capability, reducing vibration response, and enhancing structural stability. This mechanism reveals the fundamental origin of vibration attenuation and energy dissipation in feathers and provides an important theoretical basis for the design of biomimetic structures [22]. These studies indicate that bird feathers not only possess distinct hierarchical structural characteristics, but also exhibit multifunctional synergistic properties in water resistance, thermal insulation, air permeability, vibration reduction, and structural stability, making them valuable references for biomimetic textile structure design.

As an essential component of birds, feathers play a crucial role in their survival, performing multiple physiological functions such as waterproofing and thermal insulation [23,24,25]. Numerous microscopic hook-like structures are distributed along the barbules of feathers; these interlock to form smooth and strong vanes [26,27,28]. Meanwhile, birds possess abundant and dense down feathers, in which interlocking nodes exist among the filaments, maintaining effective air spaces that prevent body heat loss and thereby provide insulation [29,30]. Bird feathers consist of structures such as barbs and barbules, which are arranged in a dense and orderly manner to form a multi-level architecture, offering both thermal insulation and mechanical protection [31,32].

Furthermore, feathers distributed across different regions of a bird’s body exhibit functional differentiation in response to diverse survival requirements, and these functions impose significant constraints on their morphology [30,33,34]. For example, wing and tail feathers are primarily adapted for flight, while bristle and facial feathers serve sensory functions [35,36,37]; down feathers, mainly located in the thoracic and abdominal regions, act as the primary thermal insulation layer [38]. At the macroscopic level, feathers consist of fundamental structural components such as the rachis, barbs, and barbules [39,40]. At the microscopic level, hooklets on the barbules enable interlocking of the vane and contribute to structural stability [26,41,42], whereas plumulaceous feathers lacking a central rachis form filamentous structures with nodal connections, resulting in a loose and highly branched morphology [43]. These observations indicate that a clear structure–function relationship exists among the macroscopic form, microscopic connections, and hierarchical organization of feathers. Down, vane structures, hooklet interlocking, and filament node configurations represent typical biomimetic units suitable for translation into knitted structures. This suggests that feathers not only possess macroscopic morphological features amenable to structural translation but also exhibit micro-structural foundations that support differentiated functional expression. From a biomimetic design perspective, these structural units are closely associated with key functions such as thermal insulation and water resistance, making them representative and effective prototypes for knitted structure design.

From the above understanding of feather structure and function, it is evident that the unique structural characteristics of bird feathers can provide rich inspiration and references for the innovative design of knitted fabrics. However, although previous studies have explored the application of biomimetic structures in textile materials from various perspectives, and some have conducted comprehensive evaluations and zonal design based on multiple biomimetic knitted structures combined with thermal–moisture performance indicators and gray analysis methods, there remains further research potential in systematically extracting representative macro- and micro-structural features from bird feathers based on the relationship between functional constraints and morphological characteristics, and translating these features into manufacturable knitted structures. Moreover, at the level of apparel application, although attempts have been made to integrate structural performance with garment zonal design, most studies focus on general populations or specific usage scenarios, with a notable lack of systematic research addressing the thermal–moisture comfort needs of the elderly. Building upon the research framework proposed by Wang Jianping et al. [21] on the development and performance evaluation of biomimetic bird-feather-inspired knitted fabrics, this study further investigated the relationship between feather structure and function by selecting down feathers, feather vanes, hooklets, and fluffy feather filament node structures as biomimetic prototypes and translating them into manufacturable knitted structures. Following their general research approach, which included biomimetic structural design, thermo-moisture performance testing, gray relational optimality evaluation, and garment zonal application, this study further expanded the range of bird-feather-inspired prototypes and extended the application scenario from ski thermal pants to outdoor clothing for older adults. The aim of this study was to explore additional transformation strategies for representative bird feather structures in knitted fabric design, thereby providing references for biomimetic knitted structure development and the application of fabrics in elderly outdoor clothing.

Based on the above background, this study aims to address the following research questions:

(a) How to extract key structural features from the macroscopic morphology and microscopic mechanisms of bird feathers, and effectively translate them into knitted structures to develop biomimetic knitted designs with differentiated structural characteristics;
(b) How to analyze the differences and potential application directions of various biomimetic knitted structures in thermo-moisture performance indicators through experimental testing and comprehensive evaluation;
(c) On this basis, how to integrate the variations in heat production and sweat distribution across different body regions in the elderly, establish a mapping relationship between the performance of biomimetic knitted structures and human thermal–moisture requirements, and further develop a zonal design and application strategy for elderly outdoor clothing.

This study adopts a biomimetic perspective and designs novel knitted fabric structures based on the structural characteristics of bird feathers. In the context of accelerating global population aging, the developed fabric structures are applied to the design of outdoor clothing for the elderly, aiming to enhance both functionality and wearing comfort. Based on the macro- and micro-structural characteristics of feathers, four types of knitted structures are designed, inspired by down feathers, feather vanes, barbule hooks, and the node structures of fluffy, divergent feather filaments. Three-dimensional models of these knitted structures are constructed using the Rhino 8 modeling software and then manufactured. Subsequently, experimental equipment is used to evaluate their waterproofness, breathability, and thermal insulation properties. In addition, moisture permeability and hygroscopicity, as key indicators of fabric comfort, are also included in the testing. After completing the experiments, the data are extracted and processed to obtain performance indicators for each knitted structure. Based on these results, a gray near-optimal model is employed to comprehensively evaluate and rank the performance of the four knitted structures. Finally, considering the physiological characteristics of elderly individuals and the performance tendencies of the four knitted structures, outdoor clothing for the elderly is designed accordingly.

## 2. Materials and Methods

### 2.1. Experimental Procedure

**Figure 1:**
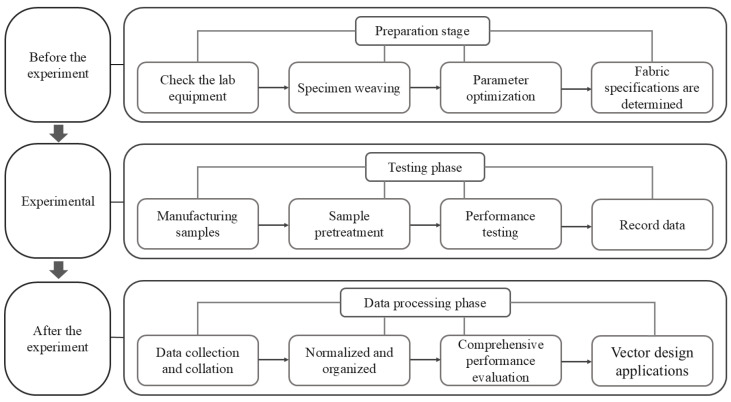
Experimental procedure.

This study builds upon the research framework proposed by Wang Jianping et al. [21] and introduces further innovations based on existing biomimetic design theories. The main innovations of this study are reflected in two aspects: the development of biomimetic structures and the zonal design strategy for elderly-oriented clothing applications. First, representative biomimetic features are extracted from the macroscopic and microscopic structural characteristics of bird feathers. Subsequently, considering the forming characteristics of knitted structures, four biomimetic knitted designs are developed, and their structural configurations are visualized using three-dimensional modeling software. The fabrics were then knitted under consistent yarn composition and machine parameters to minimize the influence of external factors on the experimental results. After fabrication, comprehensive tests were conducted on the four knitted fabrics using relevant experimental equipment, evaluating their waterproofness, thermal insulation, air permeability, moisture permeability, and hygroscopic properties. The experimental data were processed using a gray near-optimal model to provide a comprehensive evaluation of the performance of the four knitted structures. According to the physiological characteristics and outdoor activity needs of older adults, elderly outdoor clothing was designed by integrating the performance characteristics of each knitted structure, thereby providing a design basis for improving clothing comfort and functional adaptability. The overall experimental procedure is shown in Figure 1.

### 2.2. Biomimetic Design Basis and Structural Translation Representation

**Figure 2:**
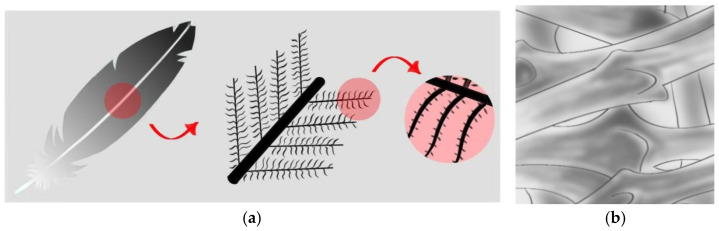
Microscopic structure of bird feathers: (**a**) interlocking mechanism of barbule hooklets; (**b**) nodal structure of fluffy, divergent feathers.

**Figure 3:**
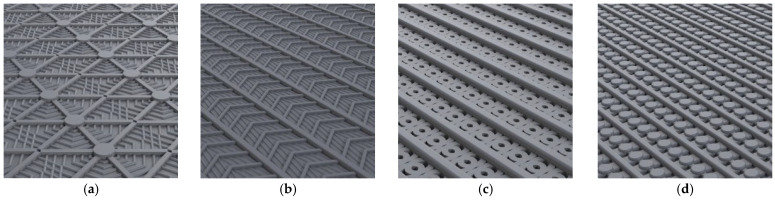
Three-dimensional effects of the biomimetic knitted structures: (**a**) imitation down feather; (**b**) imitation feather vane; (**c**) interlocking method of the imitation barbules and hooklets; (**d**) nodal structure of the filaments in imitation fluffy feathers.

In this study, four representative structural features of bird feathers were extracted, including the fluffy and divergent structure of down feathers, the hierarchical structure of feather vanes, the hook-linking mechanism of barbules, and the nodal structure of filamentous barbs in loose feathers. To clarify the basis of the biomimetic structural design, the structural characteristics of different feather regions and their translation into knitted structures were systematically summarized, as shown in Table 1. In addition, to more clearly illustrate the morphological features of the microscopic hooklet structure and feather filament node structure, these two microstructures are specifically presented in Figure 2, corresponding to Figure 2a,b, respectively. Based on these features, four biomimetic knitted structures were developed and modeled using the three-dimensional modeling software Rhino 8, with the corresponding 3D representations shown in Figure 3.

The first structure is inspired by bird down [44], as shown in Figure 3a. The overall structure exhibits a divergent form similar to the fluffy state of down, composed of strip-like elements of varying widths that mimic the barbs and filaments of down, while circular protrusions imitate the rachis structure. The second structure is innovatively designed by mimicking the overall configuration of bird feather vanes [45,46], as shown in Figure 3b. It consists of strip-like elements of different widths and lengths, representing the rachis, barbs, and barbules of feathers, respectively [47,48]. The third knitted structure is inspired by the interlocking mechanism of barbule hooklets, as shown in Figure 3c [41,49,50,51]. The widest strip-like elements represent the barbules, while the loops between them mimic the hooklet interconnections. The fourth structure is inspired by the nodal structure of fluffy feathers without a central rachis, as shown in Figure 3d. The widest elements represent the filament structures, while the circular protrusions imitate the microscopic node structures. Among these four structures, Structures 1 and 2 are developed through biomimetic design based on the macroscopic features of bird feathers, whereas Structures 3 and 4 are designed by mimicking their microscopic characteristics. By extracting the key structural features of bird feathers and integrating them with the characteristics of knitting techniques, the structures were reconstructed and translated into knitted forms. This process establishes an indirect biomimetic design pathway, thereby improving the compatibility between biomimetic concepts and knitting manufacturing processes.

### 2.3. Experimental Materials and Variable Control

To ensure the comparability among different biomimetic structural samples, a controlled variable approach was adopted in this study. The yarn type, yarn specifications, knitting equipment, and key processing parameters were kept constant, while only the knitted structure was varied. All four biomimetic bird-feather-inspired knitted fabrics were produced using yarns of identical specifications, specifically a five-ply 75 dtex spandex-blended yarn. It should be noted that the materials referred to in this study are textile fiber raw materials; no natural biological materials such as feathers were directly used in the garments. Therefore, this study represents a biomimetic design based on feather structural features rather than the direct use of natural feather materials.

During the knitting process, all four biomimetic structures were fabricated on the same machine under identical operational parameters. By standardizing the yarn materials and processing conditions, the influence of external factors on fabric performance was minimized, thereby reducing the effects of differences in yarn materials and knitting conditions on performance testing as much as possible, so that the test results primarily reflected performance differences arising from variations in knitted structural design. This provides a reliable experimental basis for subsequent comparative analyses of air permeability, thermal insulation, moisture permeability, and related properties.

### 2.4. Sample Preparation and Specification Development

Based on the construction of the three-dimensional model, knitting was carried out using a domestically produced Hengqiang computerized flat knitting machine. The knitted samples were then subjected to preliminary measurements and performance evaluations, including basic physical properties such as fabric thickness, areal density, surface flatness, density, and strength, as well as initial tests of water resistance and air permeability. The test results were used to determine whether the samples met the design expectations. If any quality issues were identified, the knitting machine parameters were adjusted and optimized accordingly based on the analysis.

Subsequently, according to the finalized sample parameters and process settings, the knitting equipment was comprehensively calibrated to ensure stability and reliability during long-term continuous operation. Yarn reserves were checked and replenished to guarantee sufficient supply and consistent quality. The working area of the knitting machine was cleaned to prevent impurities from affecting fabric quality. At the same time, the equipment was subjected to final inspection and calibration to ensure accurate execution of the designed pattern instructions during mass production.

Next, the knitting machine was operated according to the optimized process parameters and procedures to develop the fabric specifications. During the knitting process, the equipment operation was regularly inspected, and fabric quality was continuously monitored to promptly identify and address potential issues, such as yarn breakage and mechanical failures. Finally, the relevant physical properties of the fabric were measured again and recorded, including areal density, thickness, wale density, and course density.

### 2.5. Sample Pretreatment

To ensure the accuracy of experimental results and to avoid interference from dust and other impurities on the fabric surface, all samples were pretreated prior to testing. Following a standardized washing procedure, the four types of samples were placed together in a washing machine for uniform cleaning, ensuring consistent washing time and conditions for all samples. After washing, the samples were removed and laid flat on a laboratory drying rack, then air-dried naturally in a constant temperature and humidity environment to prevent fiber aging or deformation caused by high-temperature drying or direct sunlight. Once completely dry, the samples were clearly labeled by the researchers using a non-fading marker, indicating the fabric number and structural type. This preparation ensured that the samples were ready for subsequent performance tests, including water resistance, thermal insulation, air permeability, moisture permeability, and moisture absorption.

### 2.6. Experimental Equipment and Performance Indicators

This study focuses on the thermal–moisture comfort and functional protection requirements of elderly outdoor clothing, selecting water resistance, thermal insulation, air permeability, moisture permeability, and moisture absorption as the core evaluation dimensions. The corresponding performance indicators are defined as follows: water resistance, thermal insulation, air permeability, moisture permeability, and hygroscopicity. Specifically, water resistance is characterized by the degree of wetting, thermal insulation by thermal resistance and heat retention rate, air permeability by air permeability rate, moisture permeability by moisture vapor transmission rate, and moisture absorption by vertical and horizontal wicking heights.

In the performance testing stage, the fabrics were evaluated across the five dimensions—water resistance, thermal insulation, air permeability, moisture permeability, and moisture absorption—in accordance with relevant national standards. Water resistance was determined using a YG813H (manufactured in Ningbo Textile Instrument Co., Ltd., Ningbo, China) spray rating tester; thermal insulation was measured with a YG606E (manufactured in Wenzhou Fangyuan Instrument Co., Ltd., Wenzhou, China) textile thermal resistance tester; air permeability was tested using a YG461E-1 (manufactured in Ningbo Textile Instrument Co., Ltd., Ningbo, China) fabric air permeability tester; moisture permeability was assessed with a YG601H-111 (manufactured in Ningbo Textile Instrument Co., Ltd., Ningbo, China) moisture permeability tester; and moisture absorption was evaluated using a YG871 (manufactured in Wenzhou Fangyuan Instrument Co., Ltd., Wenzhou, China) capillary effect tester.

### 2.7. Test Method for Fabric Water Resistance

**Figure 4:**
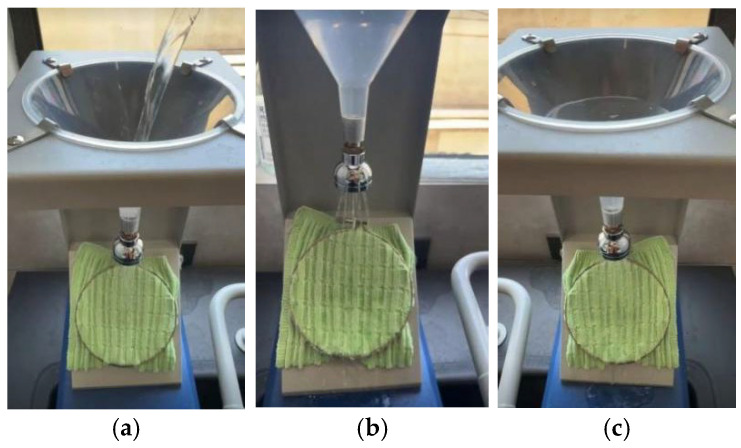
Demonstration of water resistance testing: (**a**) pouring test water into the funnel; (**b**) adding test water; (**c**) spraying.

With reference to GB/T 4745-2012 Textiles—Testing and Evaluation of Water Resistance—Spray Test, the water resistance of the fabrics was evaluated using a YG813H spray tester. For each of the four types of fabrics, four specimens measuring 18 cm × 18 cm were cut for testing. Prior to testing, the samples were conditioned under standard atmospheric conditions in accordance with GB 6529. The specimens were then mounted on a ring clamp holder, and the water resistance test was conducted as specified. The water resistance testing procedure is shown in Figure 4.

### 2.8. Test Method for Fabric Thermal Insulation Performance

**Figure 5:**
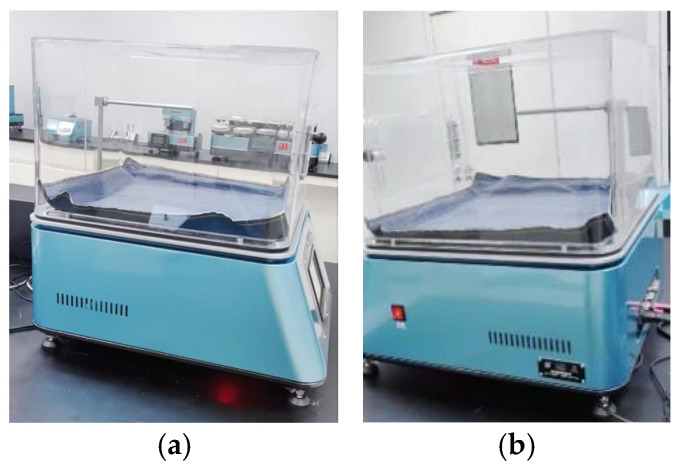
Thermal insulation testing instrument: (**a**) front view of the instrument; (**b**) rear view of the instrument.

With reference to GB/T 11048-2008 Textiles—Physiological Comfort under Steady-State Conditions—Determination of Thermal and Water Vapor Resistance, the thermal insulation performance was tested using a YG606E textile thermal resistance tester. For each of the four types of fabrics, specimens measuring 35 cm × 35 cm were prepared for testing, and three samples from different positions of each fabric were selected to ensure accuracy. The instrument used for thermal insulation performance testing is shown in Figure 5.

### 2.9. Test Method for Fabric Air Permeability

**Figure 6:**
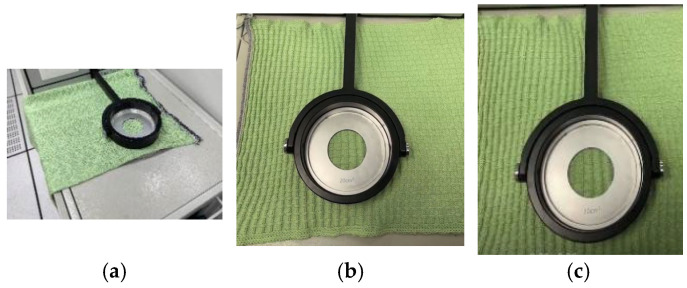
Air permeability testing process: (**a**) placing the test sample; (**b**) adjusting parameters; (**c**) starting the test.

With reference to GB/T 5453-1997 Textiles—Determination of Air Permeability of Fabrics, a YG461E-1 fully automatic air permeability tester was used to evaluate the air permeability of the fabrics based on the measured air flow rate. During testing, ten different locations on the same specimen were selected for measurement. The results from multiple measurements were recorded, and after excluding any outliers, the average value was calculated. Prior to testing, the samples were conditioned under standard atmospheric conditions in accordance with GB 6529, and the instrument was calibrated. The air permeability test was then conducted following the specified procedures. The air permeability testing procedure is shown in Figure 6.

### 2.10. Test Method for Fabric Moisture Permeability

**Figure 7:**
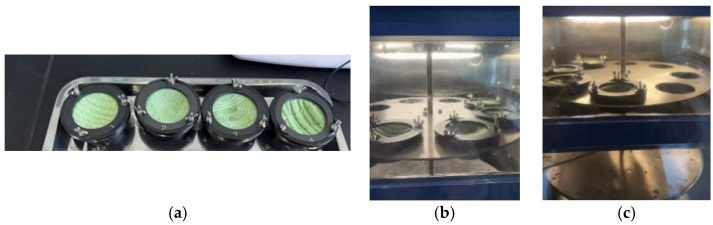
Moisture permeability testing process: (**a**) sample preparation and placement; (**b**) placing the sample into the testing instrument; (**c**) starting the test.

With reference to GB/T 12704.2-2009 Textiles—Test Methods for Water Vapor Permeability of Fabrics—Part 2: Evaporation Method, a YG601H-111 moisture permeability tester was used for evaluation. A moisture permeability cup containing distilled water at a specified temperature was sealed with the fabric specimen and placed in a controlled environment with prescribed temperature and humidity. The mass change of the cup over a certain period was measured and recorded. The moisture permeability of the fabric was then calculated based on the mass change of the cup within the given time, thereby reflecting the fabric’s moisture permeability performance. The sample preparation and testing procedure for moisture vapor permeability are shown in Figure 7.

### 2.11. Test Method for Fabric Moisture Absorption

**Figure 8:**
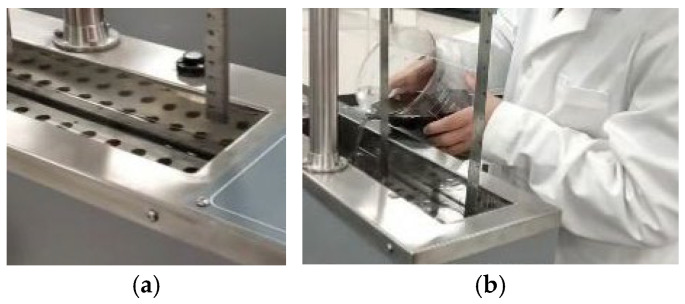
Moisture absorption testing instrument: (**a**) instrument setup; (**b**) experimental preparation.

The moisture absorption performance discussed in this study was primarily characterized by wicking height, reflecting the capillary-driven liquid water diffusion behavior within the fabric. With reference to FT/Z 01071-2008 Test Method for Capillary Effect of Textiles, a YG871 capillary effect tester was used to measure the wicking height in both the wale and course directions for four types of biomimetic knitted structures. The experimental apparatus used for moisture absorption performance testing is shown in Figure 8.

### 2.12. Comprehensive Performance Evaluation Method for Biomimetic Knitted Structures

In this study, five performance indicators of four knitted structures were experimentally tested. Since the units of these performance indicators are not consistent and it is difficult to comprehensively evaluate the overall performance of knitted fabrics based on a single indicator, it is necessary to process all the experimental data obtained. The gray relational projection method was adopted to conduct a comprehensive evaluation of fabric performance based on the data of each performance indicator. This method features a low computational workload, minimal sample size requirements, and objective and stable evaluation results [52,53]. The specific procedure for constructing the model is described as follows.

#### 2.12.1. Collection and Organization of Experimental Data

Due to the particular nature of moisture permeability data, it is necessary to perform secondary processing on the collected test data prior to model construction. Based on the experimental data, the moisture permeability rate was calculated, and the moisture permeability performance of fabrics with different structures was compared accordingly. The calculation formula is given in Equation (1). Subsequently, the categories of fabric thermal–moisture performance were defined as C_n_ (n = 1, 2, 3, 4, 5, 6, 7), and the fabric structure categories were defined as X_j_ (j = 1, 2, 3, 4). The experimental data were then classified and organized to facilitate subsequent calculations.

(1) $$\mathrm{WVT}=\frac{∆m\times 24}{A\times t}$$

where WVT—water vapor transmission per square meter per 24 h, g/(m^2^·d); ∆m—the mass difference between two weighings of the same test assembly, g; A—the area of the test specimen, m^2^; t—the test duration, h.

#### 2.12.2. Construction of the Whitening Gray Matrix

Before constructing the whitening gray matrix, it is generally necessary to first establish a gray matrix based on fabric categories and test indicators, and then construct the whitening gray matrix from it [54]. In gray system theory, a gray matrix typically refers to a matrix containing gray numbers (i.e., values with incomplete information and known only within a certain range), whereas a matrix whose elements are all definite real numbers is referred to as a white matrix. A whitening matrix is obtained by transforming the gray numbers in a gray matrix into definite values through a specific method. In this study, the performance indicators obtained from experimental tests—namely water resistance, thermal insulation, air permeability, moisture permeability, and moisture absorption—are all expressed as specific numerical values. Therefore, the step of constructing the gray matrix can be omitted, as seen in Equation (2), and the initial whitening gray matrix can be directly established based on fabric categories and test indicators; see Equation (3).

(2) $$X_{n\times m}=\left[\begin{array}{c} C_{1}\\ \vdots \\ C_{n} \end{array}\right]\left[\begin{array}{ccc} X_{11} & \cdots & X_{1m}\\ \vdots & X_{ij} & \vdots \\ X_{n1} & \cdots & X_{nm} \end{array}\right]$$

where $X_{ij}$ denotes the gray element value of the i-th performance indicator for the j-th sample; i corresponds to n, and m corresponds to j; $C_{n}$(n = 1, 2, ⋯, n) represents the fabric performance indicators, and m denotes the number of sample types.

(3) $${\bar{X}}_{n\times m}=\left[\begin{array}{c} C_{1}\\ \vdots \\ C_{n} \end{array}\right]\left[\begin{array}{ccc} {\bar{X}}_{11} & \cdots & {\bar{X}}_{1m}\\ \vdots & {\bar{X}}_{ij} & \vdots \\ {\bar{X}}_{n1} & \cdots & {\bar{X}}_{nm} \end{array}\right]$$

where ${\bar{X}}_{ij}$ represents the whitened gray element value of the i-th performance indicator for the j-th sample.

#### 2.12.3. Preprocessing of Experimental Data

During the performance testing process, each indicator corresponds to different physical meanings and measurement units, resulting in significant dimensional differences among the raw data. Direct comparison of these experimental data is therefore meaningless, as indicators with larger numerical values may dominate the final evaluation results, thereby masking the true contribution of other key properties (such as thermal insulation and moisture permeability) to the wearing comfort of elderly outdoor clothing. To enable a more intuitive comparison of the thermal–moisture performance of the four fabric structures, it is necessary to standardize each performance indicator to eliminate the influence of differing units and physical meanings. Therefore, prior to conducting a comprehensive evaluation of fabric performance, each whitened gray value—that is, the corresponding performance indicators of the four fabrics—should be normalized to the interval [0, 1] to obtain the effect measures, thereby establishing the near-optimal whitening gray matrix.

For different types of performance indicators, differentiated effect measurement formulas should be selected according to their practical significance in the design of elderly outdoor clothing. This ensures unified indicator polarity and enables a consistent evaluation across different performance dimensions. For “the-larger-the-better” indicators, such as water resistance and thermal insulation, where higher values contribute to improved protection and comfort, Equation (4) is used for positive transformation. For “moderate–optimal” indicators, Equation (5) is applied for effect measure transformation. For “the-smaller-the-better” indicators, Equation (6) is used for effect measure transformation. Through the above classification and processing, the effect measures of each fabric sample across different performance dimensions are obtained, and the near-optimal whitening gray matrix is then constructed, as shown in Equation (7).

(4) $${\bar{X^{\prime }}}_{nm}=\frac{{\bar{X}}_{ij}}{max\left\{{\bar{X}}_{ij},u_{max}\right\}}$$

(5) $${\bar{X^{\prime }}}_{nm}=\frac{min\left\{{\bar{X}}_{ij},u_{0}\right\}}{max\left\{{\bar{X}}_{ij},u_{0}\right\}}$$

(6) $${\bar{X^{\prime }}}_{nm}=\frac{min\left\{{\bar{X}}_{ij},u_{min}\right\}}{{\bar{X}}_{ij}}$$

(7) $${\bar{X^{\prime }}}_{n\times m}=\left[\begin{array}{c} C_{1}\\ \vdots \\ C_{n} \end{array}\right]\left[\begin{array}{ccc} {\bar{X^{\prime }}}_{11} & \cdots & {\bar{X^{\prime }}}_{1m}\\ \vdots & {\bar{X^{\prime }}}_{ij} & \vdots \\ {\bar{X^{\prime }}}_{n1} & \cdots & {\bar{X^{\prime }}}_{nm} \end{array}\right]$$

where in Equations (4)–(6): $u_{max}$ represents the maximum value in the i-th row of ${\bar{X}}_{ij}$, that is, the maximum value of a given category of performance indicators; $u_{o}$ represents the optimal value in the i-th row of ${\bar{X}}_{ij}$, that is, the moderate value of a given category of performance indicators; $u_{min}$ represents the minimum value in the i-th row of ${\bar{X}}_{ij}$; ${\bar{X^{\prime }}}_{ij}$ denotes the effect measure of the j-th sample with respect to the i-th indicator, and its value lies within the interval [0, 1].

#### 2.12.4. Calculation of Gray Relational Proximity

Gray relational proximity refers to an indicator used in the gray relational proximity model to measure the degree to which a given scheme approaches the optimal scheme. Based on the above steps, the relevant data obtained from experimental tests of the four knitted fabrics are substituted into Equations (3)–(6) to calculate the effect measures of the whitened gray elements, and the near-optimal whitening gray matrix ${\bar{X^{\prime }}}_{n\times m}$ is constructed accordingly. Then, the gray relational proximity ${{\bar{X}}^{\prime }}_{s}$ of each knitted structure is calculated based on the near-optimal whitening gray matrix, as shown in Equation (8).

A larger value of ${{\bar{X}}^{\prime }}_{s}$ indicates better overall performance. By comparing the gray relational proximity values of the four biomimetic knitted structures, the optimal structure in terms of comprehensive thermal–moisture performance can be determined, thereby providing a scientific basis for selecting the best-performing biomimetic knitted fabric among the four structures.

(8) $${{\bar{X}}^{\prime }}_{s}=S_{j}\left[S_{1},S_{2},\cdots ,S_{m}\right]{=S}_{j}\left[\frac{1}{n}\sum_{i=1}^{n}{{\bar{X}}^{\prime }}_{i1},\frac{1}{n}\sum_{i=1}^{n}{{\bar{X}}^{\prime }}_{i2}\ldots \frac{1}{n}\sum_{i=1}^{n}{{\bar{X}}^{\prime }}_{im}\right]$$

where $S_{j}$ represents the gray relational proximity of the j-th fabric; the larger its value, the better the overall performance of the fabric.

#### 2.12.5. Gray Relational Analysis

After constructing the gray relational proximity model and obtaining the ranking of the comprehensive proximity values for the four fabric structures, it is only possible to determine the relative overall performance of each sample. However, this does not reveal the specific contribution of each performance indicator to the overall proximity. Therefore, gray relational analysis is introduced to explore the degree of association between each performance indicator and the comprehensive proximity.

The gray relational proximity of the four knitted fabrics (No. 1–No. 4) is taken as the reference sequence $L_{0}$, and the performance test result of the j-th fabric for the i-th performance indicator is taken as the comparison sequence $L_{i}$. The performance indicators include water resistance, thermal resistance, thermal insulation rate, air permeability, moisture permeability, longitudinal wicking height, and transverse wicking height. The calculation process of the relational degree $r_{i}$ between each performance indicator and the gray relational proximity of the knitted fabrics is shown in Equations (9)–(12).

(9) $$l_{ij}=\frac{L_{ij}}{{\bar{L}}_{i}}$$

where $l_{ij}$ represents the normalized value of the test result for the j-th knitted fabric structure with respect to the i-th performance indicator; $L_{ij}$ denotes the test result of the j-th fabric for the i-th indicator; and ${\bar{L}}_{i}$ represents the normalized value of the test results for the i-th indicator across all fabrics.

(10) $$F_{ij}=\left|l_{ij}-l_{i}\right|$$

where $F_{ij}$ represents the absolute difference in the parent–subsequence for the j-th knitted fabric with respect to the i-th performance indicator, and $l_{i}$ denotes the normalized value of the gray relational proximity of the j-th knitted fabric in the reference sequence $L_{0}$.

(11) $$N_{ij}=\frac{{\left|l_{ij}-l_{i}\right|}_{min}+p{\left|l_{ij}-l_{i}\right|}_{max}}{F_{ij}+p{\left|l_{ij}-l_{i}\right|}_{max}}$$

where $N_{ij}$ is the gray relational coefficient between the i-th comparison sequence and the reference sequence; ${\left|l_{ij}-l_{i}\right|}_{min}$ is the minimum value of the difference sequence $F_{ij}$; ${\left|l_{ij}-l_{i}\right|}_{max}$ is the maximum value of the difference sequence $F_{ij}$; and p is the distinguishing coefficient, generally taken as 0.5, which is used to adjust the contrast intensity.

(12) $$r_{i}=\frac{1}{m}\sum_{j=1}^{m}N_{ij}$$

where $r_{i}$ represents the degree of association between each performance indicator and the gray relational proximity of the knitted fabrics; a larger value indicates a higher degree of association.

### 2.13. Application Method of Carrier Design

This section mainly explores the practical design applications of four knitted fabrics based on biomimetic bird feather structures and applies them to innovative designs for elderly outdoor clothing. The study focuses on the “young-old” population [55], by considering the varying requirements of different body regions in the elderly in terms of thermal insulation, moisture resistance, sweat management, and comfort, and the four biomimetic knitted structures with distinct performance characteristics were matched to corresponding garment areas to achieve a zonal design. The selection of the elderly as the target group is primarily due to their differences from younger individuals in thermoregulatory capacity, sweating behavior, and skin sensitivity, which result in more specific functional requirements for thermal insulation, moisture permeability, and overall wearing comfort. The core of this section lies in demonstrating the complete transformation process of biomimetic feather-inspired knitted structures from experimental testing to practical application. It covers the selection of design background, the study of carrier design, and the matching of fabric performance with garment functions, while also taking into account the esthetic aspects of the design carrier.

## 3. Results

### 3.1. Design Outcomes of Biomimetic Knitted Structures

**Figure 9:**
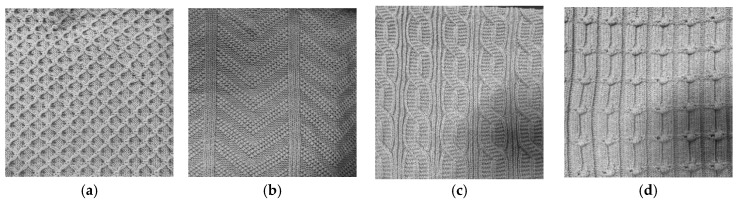
Knitted fabric effects of biomimetic structures. (**a**) Structure 1: Biomimetic down feather structure; (**b**) Structure 2: Biomimetic feather vane structure; (**c**) Structure 3: Biomimetic hook-linking barbule structure; (**d**) Structure 4: Biomimetic fluffy feather filament node structure.

**Figure 10:**
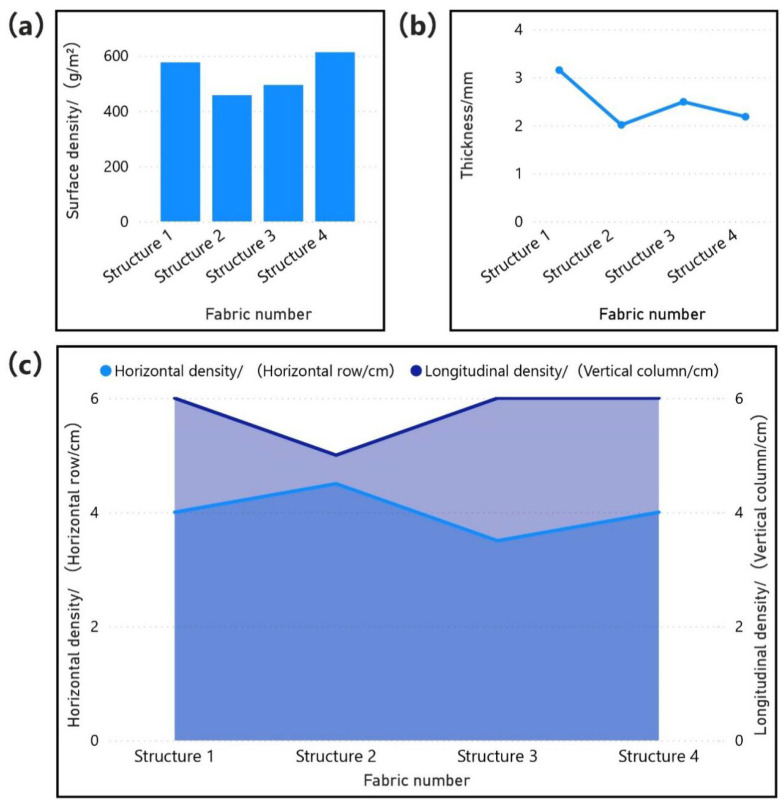
Fabric development specifications of four biomimetic fabrics. (**a**) Fabric areal density; (**b**) fabric thickness; (**c**) fabric course density and wale density.

In this study, four biomimetic knitted fabrics inspired by bird feather structures were designed and developed. Each structure draws inspiration from the unique morphology of bird feathers. The resulting knitted fabric appearances are shown in Figure 9, where the darker regions in the images are due to photographic shadows. Figure 9a presents the fabric effect of the biomimetic bird down structure, Figure 9b shows the fabric effect of the biomimetic overall feather vane structure, Figure 9c illustrates the fabric effect of the biomimetic hook-linking mechanism of feather barbules, and Figure 9d displays the fabric effect of the biomimetic fluffy feather filament node structure. The specifications of the final developed fabrics are presented in Table 2, including parameters such as areal density, thickness, and wale and course densities. The graphical results are shown in Figure 10.

### 3.2. Performance Test Results of Biomimetic Knitted Structures

**Figure 11:**
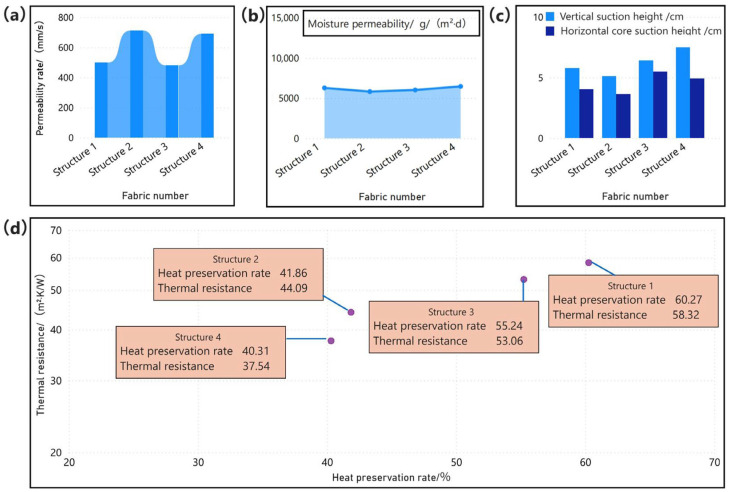
Test results of air permeability, moisture permeability, thermal insulation, and moisture absorption properties of knitted structures. (**a**) Fabric air permeability; (**b**) fabric moisture permeability; (**c**) vertical and horizontal wicking heights of the fabric; (**d**) fabric thermal resistance and insulation rate.

**Figure 12:**
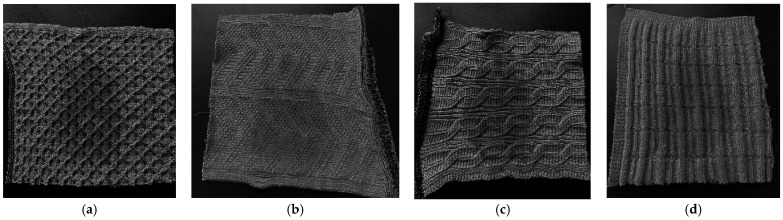
Test results of the waterproof performance of knitted structures. (**a**) Results for Structure 1; (**b**) results for Structure 2; (**c**) results for Structure 3; (**d**) results for Structure 4.

The fabrics were tested for water resistance, air permeability, thermal insulation, moisture permeability, and moisture absorption, with the detailed results presented in Table 3, and further visualized in Figure 11, while Figure 12 separately illustrates the results of the water resistance tests. Overall, the four structures exhibit differentiated performance across various performance indicators.

Structure 1, inspired by the down feather structure, exhibits a fluffy and divergent morphology. It shows relatively poor water resistance, with surface wetting and complete penetration on the reverse side. However, it demonstrates the best thermal insulation performance, with a thermal resistance of 58.324 m^2^·K/W and a heat retention rate of 60.27%, indicating excellent insulation capability. Its air permeability is 498.626 mm/s, moisture vapor transmission rate is 6278.7 g/(m^2^·d), and the vertical and horizontal wicking heights are 5.79 cm and 4.04 cm, respectively.

Structure 2, based on the feather vane structure, features a relatively compact and smooth surface. It exhibits the lowest wetting degree, with only slight surface wetting and no penetration on the reverse side. Meanwhile, its air permeability is the highest among the four structures, reaching 711.901 mm/s. However, its thermal insulation, moisture permeability, and moisture absorption performances are comparatively lower, with a thermal resistance of 44.087 m^2^·K/W, a heat retention rate of 41.86%, a moisture vapor transmission rate of 5820.3 g/(m^2^·d), and vertical and horizontal wicking heights of 5.11 cm and 3.65 cm, respectively.

Structure 3, inspired by the hook-linking mechanism of barbules, exhibits relatively balanced overall performance. It has moderate water resistance and the lowest air permeability (480.142 mm/s), while maintaining good thermal insulation performance, with a thermal resistance of 53.062 m^2^·K/W and a heat retention rate of 55.24%. In addition, its moisture vapor transmission rate is 6020.8 g/(m^2^·d), and the vertical and horizontal wicking heights are 6.42 cm and 5.50 cm, respectively, indicating relatively good moisture absorption performance.

Structure 4, based on the nodal structure of filamentous barbs in loose feathers, shows moderate water resistance and ranks second in air permeability (690.328 mm/s). Its thermal insulation performance is relatively weak, with a thermal resistance of 37.542 m^2^·K/W and a heat retention rate of 40.31%. However, the corresponding indicators for moisture permeability and moisture absorption are relatively prominent, with a moisture vapor transmission rate of 6464.9 g/(m^2^·d), a vertical wicking height of 7.51 cm, and a horizontal wicking height of 4.93 cm.

### 3.3. Comprehensive Performance Evaluation Results of Knitted Structures

After obtaining the performance indices of the four knitted fabrics through experimental testing, the gray relational optimal model was applied to analyze the data. The comprehensive evaluation results of the four knitted fabrics are shown in Table 4. According to the data in the table, the ranking of overall performance is: Structure 3 > Structure 4 > Structure 1 > Structure 2, with Structure 3 (hooked barb-like structure) exhibiting the best comprehensive performance. Further correlation analysis indicates that air permeability has the highest degree of correlation with the near-optimal performance of the knitted structures, with a correlation value of 0.826. In contrast, the longitudinal wicking property shows the lowest correlation, with a value of 0.608. Detailed results are presented in Table 5.

### 3.4. Results of Carrier Design Application

Based on the physiological characteristics of different body regions in the elderly and the functional requirements of outdoor activities, the four biomimetic fabric structures were applied in a zonal combination for elderly outdoor clothing design. The specific zoning strategy is as follows.

**Figure 13:**
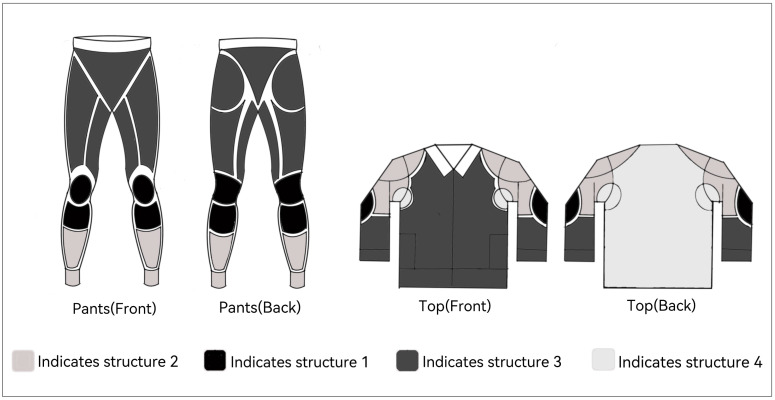
Zoned design effect of biomimetic fabric structures.

Structure 1, which exhibits excellent thermal insulation performance, was applied to the knees, elbow joints, and upper calves. Structure 4, characterized by superior moisture permeability and absorption, was used in the back area. Structure 3, with relatively good moisture permeability and absorption properties, was applied to the lower arms to reduce discomfort caused by heat and moisture accumulation. Structure 2, featuring excellent water resistance, was used in areas prone to wetting during outdoor activities, such as the lower calves, ankles, shoulders, and upper arms. For regions requiring balanced thermal–moisture performance, including the thighs, chest and abdomen, hips, and groin, Structure 3 with overall superior performance was applied. The design outcomes and the corresponding fabric structures for each zonal region are illustrated in Figure 13.

## 4. Discussion

### 4.1. Regulatory Effects of Biomimetic Knitted Structures on Thermo-Moisture Behavior

**Figure 14:**
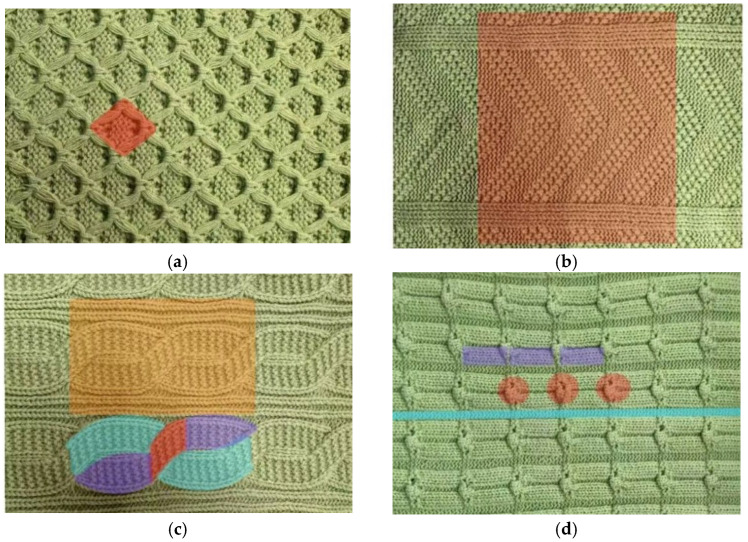
Fabric structure. (**a**) Structure 1; (**b**) Structure 2; (**c**) Structure 3; (**d**) Structure 4.

This study constructed four biomimetic knitted structures based on the structure–function characteristics of bird feathers—the structural diagram is shown in Figure 14—and systematically evaluated their waterproofness, thermal insulation, air permeability, moisture permeability, and moisture absorption properties. The results showed that different biomimetic structures exhibited distinct thermo-moisture performances. Among them, Structure 1 (down feather-inspired structure) demonstrated relatively high thermal resistance and heat retention rate; Structure 2 (barb-inspired structure) showed superior air permeability; Structure 4 (fluffy barbule node-inspired structure) exhibited relatively better moisture permeability and wicking performance; and Structure 3 (hooked barbule-inspired structure) achieved the most balanced overall performance in the gray relational proximity evaluation. These findings indicate that different hierarchical feather-inspired structures may produce different thermo-moisture transfer behaviors after being translated into knitted structures, thereby influencing fabric functional performance.

Previous studies have shown that the thermo-moisture properties of knitted fabrics are not determined by a single structural parameter, but are jointly influenced by multiple factors such as fabric structure and pore state [56,57]. Among them, Malik et al. [5] reported in their study on PES barrier fabrics that fabric air permeability is affected not only by individual yarn, fabric, and loom parameters, but also by the complex interactions among these factors. This indicates that the thermo-moisture behavior of fabrics is not governed by a single structural parameter, but rather results from the combined effects of material properties, structural characteristics, and processing factors [58].

In the present study, different performance indices did not exhibit consistent variation trends. For example, although Structure 2 showed the highest air permeability, its moisture permeability and moisture absorption performances were not prominent. Likewise, although Structure 1 had the greatest thickness, it did not exhibit the lowest moisture permeability. These findings suggest that air exchange, water vapor diffusion, and liquid moisture transport represent different thermo-moisture processes and may be affected by different structural factors [59]. Therefore, evaluating knitted fabric thermo-moisture comfort based on a single performance indicator is insufficient, and a comprehensive multi-index evaluation is necessary.

#### 4.1.1. Analysis of Air-Related Thermo-Moisture Behavior

In terms of air-related thermo-moisture behavior, Structure 1 exhibited the highest thermal resistance and heat retention rate. Based on structural observations, its fluffy and divergent morphology may form more trapped air layers, thereby reducing heat transfer efficiency. Previous studies have indicated that the thermal insulation mechanism of traditional textile materials mainly depends on air retention within the fabric structure, since air has low thermal conductivity and trapped air layers can effectively reduce heat transfer. Relatively bulky and fluffy fabric structures are generally more conducive to increasing internal air retention, thereby improving thermal resistance and insulation performance [60]. Therefore, the relatively high thermal insulation performance of Structure 1 in this study may be associated with the air retention state formed by its fluffy structure. In contrast, Structure 2 exhibited a relatively flat surface morphology, and its internal air retention may have been comparatively limited, resulting in lower thermal resistance than Structure 1. Structure 3 formed certain uneven spaces due to the interlacing loop arrangement, and thus showed better thermal insulation performance than Structure 2. Although Structure 4 contained nodal protrusion structures, the continuity of its internal air layer may have been insufficient, leading to relatively weaker thermal insulation performance.

In terms of air permeability, noticeable differences were also observed among the four structures. Previous studies have shown that thermal properties, diffusion behavior, and air and water vapor permeability are jointly influenced by raw material types and fabric structural parameters [61]. Therefore, the differences in air permeability among the biomimetic structures in this study may also be related to factors such as fabric structure and other associated parameters.

#### 4.1.2. Analysis of Moisture Transfer Behavior

**Figure 15:**
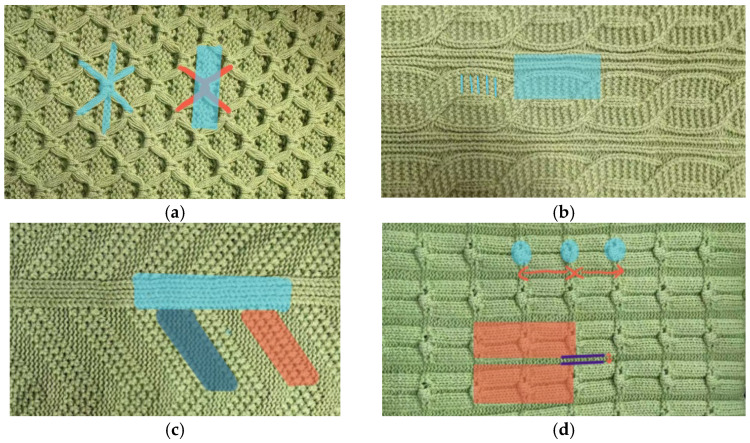
Analysis of fabric organizational Structure. (**a**) Structure 1; (**b**) Structure 3; (**c**) Structure 2; (**d**) Structure 4. (Different colors in each figure represent the analysis of the fabric organizational structure to illustrate the characteristics of the fabric structure, while the arrows indicate the distance.)

The moisture permeability results showed that Structure 4 exhibited the highest moisture permeability, while Structure 2 showed the lowest. Previous studies have indicated that water vapor transport is related to the internal spatial configuration and pore connectivity of fabrics [59]. Based on structural observations, the organizational structure analysis diagrams of the four knitted structures are shown in Figure 15. Structure 4 consisted of strip-like structures and nodal protrusions, which may have formed relatively continuous spaces for water vapor diffusion, thereby contributing to its higher moisture permeability. Although Structure 1 had a relatively large thickness, its fluffy and divergent structure may have created more open spaces, resulting in comparatively high moisture permeability as well. In contrast, Structure 2 exhibited a relatively flat morphology with fewer internal open spaces, which may explain its lower moisture permeability.

It should be noted that moisture permeability did not show the same variation trend as air permeability. For example, although Structure 2 exhibited the highest air permeability, it showed the lowest moisture permeability. This suggests that air exchange capacity cannot be directly equated with water vapor diffusion capability, as the two processes may be influenced by different structural factors [59].

In terms of moisture absorption performance, wicking behavior mainly reflects the capillary diffusion ability of liquid water within fabrics. Previous studies have indicated that yarn arrangement and fabric interlacing structures can affect capillary geometry and further alter the diffusion behavior of liquid moisture within fabrics. In yarn interlacing regions, liquid flow may be redistributed in different directions, thereby influencing wicking height and diffusion directionality [62]. Based on the structural observations in this study, the strip-like structures and nodal arrangement in Structure 4 exhibited certain directional characteristics along the longitudinal direction, which may facilitate liquid moisture diffusion along the fabric direction. In contrast, Structure 3 showed relatively better transverse wicking performance due to the transverse arrangement formed between loops. Compared with these structures, Structure 2 exhibited a relatively flat and compact morphology, which may not favor liquid moisture diffusion within the fabric, resulting in lower wicking performance.

In this study, moisture absorption performance also did not exhibit the same variation trend as air permeability. For example, although Structure 2 showed the highest air permeability, its wicking performance was relatively low, whereas Structure 4 demonstrated superior moisture absorption and moisture permeability but relatively weaker thermal insulation performance. These findings further suggest that different thermo-moisture behaviors in knitted fabrics do not necessarily exhibit simple linear relationships, but may instead be jointly influenced by multiple factors [63].

#### 4.1.3. Multi-Performance Coordination and Comprehensive Performance Evaluation

In terms of comprehensive performance evaluation, Structure 3 (hooked barbule-inspired structure) achieved the highest gray relational proximity value, exhibiting relatively balanced thermo-moisture performance characteristics. Its advantage was mainly reflected in the relative balance among thermal insulation, moisture permeability, and moisture absorption properties, rather than in the superiority of a single performance indicator. The gray relational analysis further showed that air permeability had the highest correlation with the comprehensive proximity value, indicating that air permeability had a relatively strong influence on overall thermo-moisture comfort within the evaluation system established in this study.

**Figure 16:**
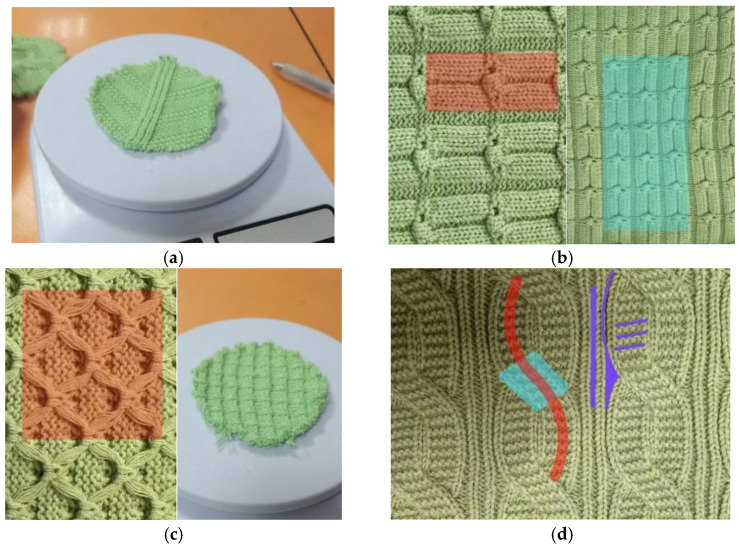
Observational analysis of fabric organizational structures and thickness characteristics. Differences were observed among the structures in terms of thickness characteristics and organizational morphology, and different colors in each figure were used to represent the characteristics of the fabric organizational structures. (**a**) Structure 2; (**b**) Structure 4; (**c**) Structure 1; (**d**) Structure 3.

To further analyze the reasons for the differences in thermo-moisture performance among different biomimetic knitted structures, the fabric thickness and organizational structures were observed and analyzed, as shown in Figure 16. Differences were observed among the structures in terms of organizational morphology, pore distribution, and thickness characteristics, and these structural features may affect airflow and thermo-moisture transfer behavior.

These findings further suggest that different biomimetic structures tend to exhibit advantages in certain specific performance indices, while it remains difficult to simultaneously optimize all thermo-moisture properties. Therefore, compared with pursuing the optimization of a single property, achieving multi-performance balance through structural coordination may be more suitable for functional clothing design [64].

### 4.2. Implications of Biomimetic Knitted Structures for Elderly Outdoor Clothing Design

From an application perspective, this study further explored the zonal application of different biomimetic structures in elderly outdoor clothing design. Previous studies have shown that elderly individuals differ from younger populations in thermoregulation, regional sweat distribution, and thermo-moisture adaptation capacity [1,2,3,4]. Therefore, compared with conventional single-fabric designs, functional zoning based on thermo-moisture requirements may be more suitable for elderly outdoor clothing.

Based on the experimental results, different biomimetic knitted structures with distinct performance characteristics were assigned to different functional regions. For example, Structure 1, which exhibited relatively better thermal insulation performance, may be suitable for cold-sensitive areas such as the knees and elbow joints; Structure 4, which showed superior moisture permeability and moisture absorption properties, may be more appropriate for sweat-prone regions such as the back; and Structure 3, which demonstrated relatively balanced overall performance, may be applicable to areas with comprehensive thermo-moisture demands such as the chest, abdomen, and hip regions. The specific results are presented in Table 6. This design strategy does not directly establish a one-to-one correspondence between feather structures and human body regions, but rather is based on the matching relationship between fabric performance characteristics and localized thermo-moisture requirements of the human body [21]. Therefore, this study not only focused on the thermo-moisture performance of biomimetic structures themselves, but also further explored the application potential of biomimetic knitted structures in functional clothing design for elderly populations.

### 4.3. Comparison with Previous Biomimetic Textile Studies

Compared with the study conducted by Wang Jianping et al. [21] on biomimetic feather-inspired knitted structures, both studies observed significant differences in thermo-moisture performance among different biomimetic structures. For example, the barb-inspired structures in both studies exhibited relatively high air permeability, while some groove-like or interlocking structures demonstrated comparatively better wicking performance. These findings suggest that hierarchical feather-inspired structures may influence the thermo-moisture behavior of fabrics after being translated into knitted structures [61].

However, it should be noted that noticeable differences still existed in the specific performance values reported in the two studies. For example, the air permeability of Structure 2 in the present study was significantly higher than that reported in their research. This indicates that even when similar biomimetic prototypes are adopted, differences in yarn specifications, knitting parameters, and structural translation methods may further alter the internal pore state and thermo-moisture behavior of fabrics. Therefore, the performance of feather-inspired biomimetic structures is not determined solely by the biomimetic prototype itself, but is jointly influenced by multiple factors including materials, fabric structures, and processing parameters [61].

In addition, previous studies on biomimetic porous fabrics have also indicated that structural design may influence liquid moisture transport and water vapor diffusion behavior in fabrics [65,66]. Although the material systems and structural forms used in different studies were not entirely consistent, these studies collectively suggest that fabric structural characteristics may be associated with thermo-moisture behavior. The present study further verified the differences in thermo-moisture behavior among different biomimetic knitted structures from the perspective of knitted structural design.

### 4.4. Significance of Biomimetic Design and Sustainability Discussion

Based on the above analysis of performance and structural applications, this study can be further discussed from the perspective of biomimetic design. Taking the hierarchical structure of bird feathers as the biomimetic prototype, this study translates structural features such as down feathers, feather vanes, barbule hooks, and filament nodes into knitted fabric designs, and systematically evaluates their performance through tests of water resistance, thermal insulation, air permeability, moisture permeability, and moisture absorption. The results indicate that the four biomimetic knitted structures exhibit differentiated performance across these indicators, demonstrating that feather structural characteristics can provide valuable references for knitted fabric design.

From a biomimetic design perspective, the significance of this study lies in the translation of both macroscopic morphology and microscopic features of bird feathers into manufacturable knitted structures. Unlike approaches that merely mimic biological appearance, this study focuses on the structural translation of feather features into textile constructions and their corresponding effects on thermal–moisture performance. This process provides experimental support for the application of biomimetic structures in functional knitted fabric design.

The results show that the down-inspired structure performs well in thermal insulation, the vane-inspired structure exhibits advantages in water resistance and air permeability, the filament-node-inspired structure demonstrates superior moisture permeability and absorption, and the hook-linked structure achieves the best overall performance. These findings indicate that different structures exhibit distinct performance emphases, and a single structure cannot simultaneously satisfy all functional requirements. Therefore, the application of multiple biomimetic structures in a zonal garment design is proposed based on the experimental results.

Compared with existing biomimetic textile studies, the uniqueness of this work lies in three aspects. First, it systematically compares the performance differences in four types of feather-inspired macro–micro structures in knitted fabrics. Second, it integrates the gray relational optimization model with the thermal–moisture requirements of elderly individuals to establish a comprehensive performance evaluation framework tailored to this population. Third, it proposes a full-body zonal design strategy based on performance differentiation, rather than focusing on a single body region.

From the perspective of sustainable development, biomimetic design emphasizes performance enhancement through structural optimization, thereby reducing reliance on energy-intensive finishing processes or complex material systems. In this study, performance variation is primarily achieved through adjustments in knitted structures, which may help reduce the use of chemical treatments and thus lower environmental impact.

In summary, the contributions of this study are threefold. First, it translates the hierarchical structure of bird feathers into knitted fabric design, expanding the application of feather-inspired biomimetics in textile structural design. Second, it clarifies the performance tendencies of different biomimetic structures through experimental comparison, providing a basis for the zonal design of elderly outdoor clothing. Third, it proposes a design pathway for functional knitted fabrics that can be further validated in future studies.

## 5. Conclusions

This study constructed four biomimetic knitted structures based on the “function-constrained morphology” characteristics of bird feathers, focusing on typical structural units including down feathers, barbs, hooked barbules, and fluffy barbule nodes. Through waterproofness, thermal insulation, air permeability, moisture permeability, and moisture absorption tests, the thermo-moisture behavior characteristics of different biomimetic structures were systematically analyzed. On this basis, comprehensive performance was evaluated using the gray relational proximity model and gray relational analysis, and their zonal application in elderly outdoor clothing was further explored.

The experimental results indicate that the four biomimetic structures exhibit distinct performance characteristics. The down-inspired structure, due to its fluffy characteristics, may increase the amount of still air within the fabric, thereby enhancing thermal resistance and insulation rate, with a thermal resistance of 58.324 m^2^·K·W^−1^, the vane-inspired structure performs well in terms of wetting degree and air permeability, with an air permeability of 711.901 mm/s, the filament-node-inspired structure shows relatively high values in moisture permeability and capillary wicking height, with a moisture permeability of 6464.9 g/(m^2^·d), and the barbule-inspired structure achieves the highest overall performance evaluation, with a relative closeness of 0.8812, exhibiting a relatively balanced performance profile. Gray relational analysis further reveals that air permeability has a relatively strong influence on the overall performance evaluation. The study also found that different thermo-moisture performance indicators did not exhibit simple linear relationships. This suggests that the thermo-moisture behavior of knitted fabrics is not determined by a single structural parameter, but may instead be jointly influenced by multiple factors such as fabric morphology and pore structure.

Based on the performance differences described above, this study further explored the zonal application of the four biomimetic knitted structures in elderly outdoor clothing. This application strategy does not directly establish a correspondence between feather functions and human body regions, but instead assigns fabric structures with different performance characteristics to areas with specific functional requirements according to the testing results of the knitted structures, thereby improving localized functional adaptability of the clothing.

From both theoretical and practical perspectives, the innovations of this study are reflected in two aspects. First, a knitted structure translation approach based on the function-constrained morphology of bird feathers was proposed. Second, the target research population was extended to the elderly population, and a zonal design strategy for elderly clothing was proposed by integrating the performance characteristics of knitted structures with functional clothing requirements.

## 6. Limitations

It should be noted that this study still has certain limitations, which also indicate issues that require further investigation in future research. First, the present study primarily interpreted the thermo-moisture performance differences among biomimetic knitted structures based on structural observations and performance testing results. Quantitative characterization of structural parameters such as porosity, pore connectivity, pore size distribution, and air-layer continuity has not yet been conducted; therefore, the relationships between structural parameters and performance indicators still require further clarification. Second, in order to emphasize the influence of structural differences on performance, yarn materials, yarn specifications, and major knitting parameters were unified in this study. However, variations in yarn materials, knitting density, and finishing treatments may alter the thermo-moisture performance of biomimetic structures. Future studies could therefore conduct multifactor coupling experiments to further optimize structural design and knitting processes. Finally, the zonal application strategies proposed in this study were mainly inferred from fabric performance testing results and have not yet been validated through real wearing trials or dynamic environmental simulations. Accordingly, future research may focus on quantitative structural parameter analysis, multifactor process coupling, and validation under actual wearing environments to further improve the application reliability of biomimetic knitted structures in functional clothing.

## Figures and Tables

**Table 1:** Translation relationship between biomimetic bird feather structures and knitted structures.

Biomimetic Prototype	Biological Structural Characteristics	Basis for Structural Feature Extraction	Knitted Structure Translation Strategy	Corresponding Knitted Structure
Down Feather	Feather branches are divergently distributed, with a loose structure and numerous pores	Divergent arrangement + porous structure	A divergent strip-like structure combined with raised elements was adopted to increase internal air retention space within the fabric	Structure 1
Feather Vane	Feather branches are arranged hierarchically, with a relatively flat and continuous structure	Hierarchical arrangement + surface continuity	A multilayered strip-like structure was adopted to form a relatively flat and continuous fabric surface	Structure 2
Hooklet	Microscopic hook-like interlocking structures connect adjacent feather branches	Hooklet structure + nodal connection	An interlooped knitted structure was adopted to form nodal connection features and enhance structural connectivity	Structure 3
Fluffy Feather Filament Node	Feather filaments extend and intersect to form nodal structures with strong spatial characteristics	Strip extension + nodal distribution	A strip-like knitted structure combined with raised nodal elements was adopted	Structure 4

**Table 2:** Fabric development specifications.

Fabric No.	Appearance Structure	Areal Density (g/m^2^)	Thickness (mm)	Course Density	Wale Density
Structure 1	Biomimetic down structure	576	3.15	4 courses/cm	6 wales/cm
Structure 2	Biomimetic feather vane structure	457	2.01	4.5 courses/cm	5 wales/cm
Structure 3	Biomimetic hook-linking barbule structure	494	2.49	3.5 courses/cm	6 wales/cm
Structure 4	Biomimetic feather filament node structure	613	2.18	4 courses/cm	6 wales/cm

**Table 3:** Test results of four performance indicators of knitted structures (excluding water resistance).

Sample Name	Air Permeability (mm/s)	Moisture Permeability (g/(m^2^·d))	Thermal Insulation Performance		Wicking Height (cm)	
			Thermal Resistance (m^2^·K·W^−1^)	Thermal Insulation Rate (%)	Longitudinal Direction	Transverse Direction
Structure 1	498.626	6278.7	58.324	60.27	5.79	4.04
Structure 2	711.901	5820.3	44.087	41.86	5.11	3.65
Structure 3	480.142	6020.8	53.062	55.24	6.42	5.50
Structure 4	690.328	6464.9	37.542	40.31	7.51	4.93

**Table 4:** Comprehensive performance evaluation results of fabrics.

	Structure 1	Structure 2	Structure 3	Structure 4
Comprehensive evaluation results	0.8629	0.7825	0.8812	0.8631

**Table 5:** Gray relational analysis.

	Air Permeability	Moisture Permeability	Thermal Resistance	Thermal Insulation Rate	Vertical Wicking Height	Horizontal Wicking Height
Correlation degree	0.826	0.630	0.626	0.791	0.608	0.775

**Table 6:** Matching of physiological requirements and fabric structures.

Body Parts	Characteristics	Fabric Structure
Knees and elbow joints	Easily feels cold	Structure 1 (excellent thermal insulation performance)
Back	Prone to sweating	Structure 4 (excellent moisture permeability and moisture absorption performance)
Forearms	Relatively prone to sweating	Structure 3 (relatively good moisture permeability and absorption performance)
Lower calves, ankles, shoulders, and upper arms	Easily gets wet	Structure 2 (excellent waterproof performance)
Upper calves	Easily feels cold	Structure 1 (excellent thermal insulation performance)
Thighs, chest and abdomen, hips, and groin	Based on fabric thermal and moisture properties	Structure 3 (excellent overall performance with certain thermal insulation capability)

## Data Availability

Data are available upon request.

## References

[1] Saxon S.V., Etten M.J., Perkins E.A. (2021). Physical Change and Aging: A Guide for Helping Professions.

[2] Smith C.J., Alexander L.M., Kenney W.L. (2013). Nonuniform, age-related decrements in regional sweating and skin blood flow. Am. J. Physiol.-Regul. Integr. Comp. Physiol..

[3] Amano T., Ichinose-Kuwahara T., Ueda H., Kondo N., Wang H., Inoue Y. (2026). Biological aging and sex differences in cholinergic sweating: From young adults to the elderly in their 80s and beyond. Am. J. Physiol.-Regul. Integr. Comp. Physiol..

[4] Preston J., Biddell B. (2021). The physiology of ageing and how these changes affect older people. Medicine.

[5] Malik S.A., Kocaman R.T., Kaynak H.K., Gereke T., Aibibu D., Babaarslan O., Cherif C. (2017). Analysis and prediction of air permeability of woven barrier fabrics with respect to material, fabric construction and process parameters. Fibers Polym..

[6] Kim H.-A. (2021). Moisture vapor permeability and thermal wear comfort of ecofriendly fiber-embedded woven fabrics for high-performance clothing. Materials.

[7] Teyeme Y., Malengier B., Tesfaye T., Vasile S., Van Langenhove L. (2020). Comparative analysis of thermophysiological comfort-related properties of elastic knitted fabrics for cycling sportswear. Materials.

[8] Yang Y., Yu X., Chen L., Zhang P. (2021). Effect of knitting structure and yarn composition on thermal comfort properties of bi-layer knitted fabrics. Text. Res. J..

[9] Tamborini M. (2024). From biomimicry to robotic co-creation: Rethinking the boundaries between nature and technology. Bioinspir. Biomim..

[10] Dixit S., Stefańska A. (2023). Bio-logic, a review on the biomimetic application in architectural and structural design. Ain Shams Eng. J..

[11] Rose J.B.R., Natarajan S.G., Gopinathan V. (2021). Biomimetic flow control techniques for aerospace applications: A comprehensive review. Rev. Environ. Sci. Bio/Technol..

[12] Schmidt A.-M., Schmelzeisen D., Gries T. (2022). 4D-textiles: Development of bistable textile structures using rapid prototyping and the bionic approach. Rapid Prototyp. J..

[13] Eadie L., Ghosh T.K. (2011). Biomimicry in textiles: Past, present and potential. An overview. J. R. Soc. Interface.

[14] Shateri-Khalilabad M., Yazdanshenas M.E. (2013). Fabrication of superhydrophobic, antibacterial, and ultraviolet-blocking cotton fabric. J. Text. Inst..

[15] Wu M., Shao Z., Zhao N., Zhang R., Yuan G., Tian L., Zhang Z., Gao W., Bai H. (2023). Biomimetic, knittable aerogel fiber for thermal insulation textile. Science.

[16] Jayamaha H., Park K., Shepherd L.M. (2025). Ultrablack wool textiles inspired by hierarchical avian structure. Nat. Commun..

[17] Singh A.V., Rahman A., Kumar N.S., Aditi A., Galluzzi M., Bovio S., Barozzi S., Montani E., Parazzoli D. (2012). Bio-inspired approaches to design smart fabrics. Mater. Des. (1980–2015).

[18] Hoffmann K.A., Chen T.G., Cutkosky M.R., Lentink D. (2023). Bird-inspired robotics principles as a framework for developing smart aerospace materials. J. Compos. Mater..

[19] Murayama Y., Nakata T., Liu H. (2021). Flexible flaps inspired by avian feathers can enhance aerodynamic robustness in low Reynolds number airfoils. Front. Bioeng. Biotechnol..

[20] Tu R., Delplanche R.A., Tobalske B.W., Inman D.J., Sodano H.A. (2024). 3D printed feathers with embedded aerodynamic sensing. Bioinspiration Biomim..

[21] Wang J., Miao M., Shen D., Yao X. (2022). Development and Performance Evaluation of Knitted Fabrics with Biomimetic Bird Feather Structures. J. Text. Res..

[22] Deng K., Rajabi H., Kovalev A., Schaber C.F., Dai Z., Gorb S.N. (2023). The role of vanes in the damping of bird feathers. J. Bionic Eng..

[23] Terrill R.S., Shultz A.J. (2023). Feather function and the evolution of birds. Biol. Rev..

[24] Muzio F.M., Rubega M.A. (2024). What do we really know about the water repellency of feathers?. J. Avian Biol..

[25] Mueller J., Gibson L. (2023). Structure and mechanics of water-holding feathers of Namaqua sandgrouse (*Pterocles namaqua*). J. R. Soc. Interface.

[26] Sullivan T.N., Chon M., Ramachandramoorthy R., Roenbeck M.R., Hung T.T., Espinosa H.D., Meyers M.A. (2017). Reversible attachment with tailored permeability: The feather vane and bioinspired designs. Adv. Funct. Mater..

[27] Song W., Mu Z., Zhang Z., Wang Y., Hu H., Ma Z., Huang L., Wang Z., Zhang B., Li Y. (2021). Cross-scale biological models of species for future biomimetic composite design: A review. Coatings.

[28] Muzio F.M., Rubega M.A. (2025). Differences in Microstructure Morphology Results in Variable Wettability Across Feather Types in a Terrestrial Bird Species. J. Morphol..

[29] Pap P.L., Osváth G., Daubner T., Nord A., Vincze O. (2020). Down feather morphology reflects adaptation to habitat and thermal conditions across the avian phylogeny. Evolution.

[30] Barve S., Ramesh V., Dotterer T.M., Dove C.J. (2021). Elevation and body size drive convergent variation in thermo-insulative feather structure of Himalayan birds. Ecography.

[31] Zhuge Y., Liu F. (2024). Recent progress of bionic hierarchical structure in the field of thermal insulation protection. J. Bionic Eng..

[32] McCafferty D.J., Pandraud G., Gilles J., Fabra-Puchol M., Henry P.Y. (2018). Animal thermoregulation: A review of insulation, physiology and behaviour relevant to temperature control in buildings. Bioinspiration Biomim..

[33] Kiat Y., O’Connor J.K. (2024). Functional constraints on the number and shape of flight feathers. Proc. Natl. Acad. Sci. USA.

[34] Lin P.-Y., Huang P.-Y., Lee Y.-C., Ng C.S. (2022). Analysis and comparison of protein secondary structures in the rachis of avian flight feathers. PeerJ.

[35] Sibley D.A. (2020). What It’s Like to Be a Bird: From Flying to Nesting, Eating to Singing—What Birds Are Doing, and Why.

[36] McWilliams S., Clarke J.A., MacDougall-Shackleton E., MacDougall-Shackleton S., Bonier F., Eliason C. (2021). What Is a Bird?: An Exploration of Anatomy, Physiology, Behavior, and Ecology.

[37] Jenni L., Winkler R. (2020). Moult and Ageing of European Passerines.

[38] Amaya-Mejia W., Lim S., Ma L., Shultz A.J., Yeh P. (2025). Feather macrostructure is marginally correlated with temperature range but not urbanization across California. Sci. Rep..

[39] Alibardi L. (2017). Cornification, morphogenesis and evolution of feathers. Protoplasma.

[40] Kiere L.M., López-Michelena A., Osorio-Beristain M., Sorani V., Navarro-Sigüenza A.G., Sánchez-González L.A. (2023). Proximity to metal mining is related to aspects of feather coloration but not fluctuating asymmetry in the Russet-crowned Motmot (*Momotus mexicanus*) in south-central Mexico. Ibis.

[41] Coddington C.P., Dove C.J., Luther D.A. (2022). Microscopic analysis of the plumulaceous feather characteristics of Cathartiformes and Accipitriformes in North America. J. Raptor Res..

[42] Matloff L.Y., Chang E., Feo T.J., Jeffries L., Stowers A.K., Thomson C., Lentink D. (2020). How flight feathers stick together to form a continuous morphing wing. Science.

[43] Hendrickx-Rodriguez S., Lentink D. (2025). The feather’s multi-functional structure across nano to macro scales inspires hierarchical design. J. R. Soc. Interface.

[44] Chen C.-K., Chang Y.-M., Jiang T.-X., Yue Z., Liu T.-Y., Lu J., Yu Z., Lin J.-J., Vu T.-D., Huang T.-Y. (2024). Conserved regulatory switches for the transition from natal down to juvenile feather in birds. Nat. Commun..

[45] Ksepka D.T. (2020). Feathered dinosaurs. Curr. Biol..

[46] Świsłowski P., Hebda G., Zinicovscaia I., Chaligava O., Isinkaralar O., Isinkaralar K., Rajfur M. (2025). I believe I can fly… but in polluted air, why? Bird feathers as an example of environmental contaminant monitoring. Sci. Total Environ..

[47] Zhang Y.-Y., Tang J.-W., Wang Y., Wang S. (2025). Medulla-free barb rami highlight the morphological diversity of early feathers. Zool. Res..

[48] Osvath G., Vincze O., David D.-C., Nagy L.J., Lendvai A.Z., Nudds R.L., Pap P.L. (2020). Morphological characterization of flight feather shafts in four bird species with different flight styles. Biol. J. Linn. Soc..

[49] Lopatka A. (2020). Barbs of a feather lock together. Phys. Today.

[50] Moulzir M., Hazart D., Delhomme B., Oheim M., Ricard C. (2026). Light as a feather: A novel approach for whole-feather microstructure imaging through tissue clearing and autofluorescence imaging. Ann. Anat.-Anat. Anz..

[51] Jeffries L., Lentink D. (2020). Design principles and function of mechanical fasteners in nature and technology. Appl. Mech. Rev..

[52] Dai M., Tu Y. (2020). Grey Relational Near-Optimal Evaluation of Wearing Comfort for New-Type Wadding Materials and Traditional Wadding Materials. Wool Text. J..

[53] Wu G., Cheng H. (2018). Performance Evaluation of Knitted T-shirt Fabrics Based on the Grey Relational Near-Optimal Model. Wool Text. J..

[54] Wang C., Chen M., Hou L., Fan Z., Li X. (2025). Research and Development of Graphene-Based Antiviral Protective Fabrics. Synth. Fiber China.

[55] Xu Y., Shen Y. (2022). Study on the Group Characteristics and Intergenerational Responsibility of Urban “New Elderly”. Acad. J. Zhongzhou.

[56] Bedek G., Salaün F., Martinkovska Z., Devaux E., Dupont D. (2011). Evaluation of thermal and moisture management properties on knitted fabrics and comparison with a physiological model in warm conditions. Appl. Ergon..

[57] Song X., Wei L. (2013). The Research on Wearable Comforts of Formal Terry Fabric. J. Yancheng Inst. Technol. (Nat. Sci. Ed.).

[58] Kumar R., Saxena A. (2025). A Study on Thermal Comfort Properties in Knit Fabrics Made from Cattail and Cotton Blends. Fibers Polym..

[59] Su Y., Fan Y., Liu G., Tian M., Li J. (2023). A review on sustainable method to evaluate heat and moisture transfer in clothing material. Sustainability.

[60] Venkataraman M., Mishra R., Kotresh T.M., Militky J., Jamshaid H. (2016). Aerogels for thermal insulation in high-performance textiles. Text. Prog..

[61] Onofrei E., Rocha A.M., Catarino A. (2011). The Influence of Knitted Fabrics’ Structure on the Thermal and Moisture Management Properties. J. Eng. Fibers Fabr..

[62] Roy M.D., Chattopadhyay R., Sinha S.K. (2018). Wicking Performance of Profiled Fibre Part B: Assessment of Fabric. J. Inst. Eng. Ser. E.

[63] Jhanji Y., Gupta D., Kothari V.K. (2017). Effect of fibre, yarn and fabric variables on heat and moisture transport properties of plated knit. Indian J. Fibre Text. Res..

[64] Qian J., Li Y., Xiang Z.L., Cai H.M., Zhang P.H. (2022). Effect of weave structure and yarn fineness on the coolness and thermal-wet comfort properties of woven fabric. Text. Res. J..

[65] Zuo L.J., Zhang Q., Tu J.N., Nie M.T. (2025). Study on the moisture permeability of warp-knitted Jacquard shoe upper material based on CFD. Sci. Rep..

[66] Tian X., Li J.N., Li L. (2024). Bioinspired green fabricating design of ultra-breathable and moisture wicking fabric via a sustainable route. J. Clean. Prod..

